# Determination of the μ-Conotoxin PIIIA Specificity Against Voltage-Gated Sodium Channels from Binding Energy Calculations

**DOI:** 10.3390/md16050153

**Published:** 2018-05-07

**Authors:** Fangling Chen, Wenxin Huang, Tao Jiang, Rilei Yu

**Affiliations:** 1 Key Laboratory of Marine Drugs, Chinese Ministry of Education, School of Medicine and Pharmacy, Ocean University of China, Qingdao 266003, China; Chenfangling0410@hotmail.com (F.C.); annie95116@gmail.com (W.H.); jiangtao@ouc.edu.cn (T.J.); 2 Laboratory for Marine Drugs and Bioproducts, Qingdao National Laboratory for Marine Science and Technology, Qingdao 266003, China

**Keywords:** homology modeling, molecular docking, sodium channel, μ-conotoxin, MD simulation, binding affinity, MMGB/SA, umbrella sampling

## Abstract

Voltage-gated sodium (Na_V_) channels generate and propagate action potentials in excitable cells, and several Na_V_ subtypes have become important targets for pain management. The μ-conotoxins inhibit subtypes of the Na_V_ with varied specificity but often lack of specificity to interested subtypes. Engineering the selectivity of the μ-conotoxins presents considerable complexity and challenge, as it involves the optimization of their binding affinities to multiple highly conserved Na_V_ subtypes. In this study, a model of Na_V_1.4 bound with μ-conotoxin PIIIA complex was constructed using homology modeling, docking, molecular dynamic simulations and binding energy calculations. The accuracy of this model was confirmed based on the experimental mutagenesis data. The complex models of PIIIA bound with varied subtypes of Na_V_1.x (x = 1, 2, 3, 5, 6, 7, 8, or 9) were built using Na_V_1.4/PIIIA complex as a template, and refined using molecular dynamic simulations. The binding affinities of PIIIA to varied subtypes of Na_V_1.x (x = 1 to 9) were calculated using the Molecular Mechanics Generalized Born/Surface Area (MMGB/SA) and umbrella sampling, and were compared with the experimental values. The binding affinities calculated using MMGB/SA and umbrella sampling are correlated with the experimental values, with the former and the latter giving correlation coefficient of 0.41 (R^2^) and 0.68 (R^2^), respectively. Binding energy decomposition suggests that conserved and nonconserved residues among varied Na_V_ subtypes have a synergistic effect on the selectivity of PIIIA.

## 1. Introduction

The μ-conotoxins are short snail peptide toxins specifically targeting subtypes of Na_V_ and are considered as important drug leads. The first step for engineering the specificity of μ-conotoxins is understanding of the molecular interaction mechanism between the μ-conotoxins and varied subtypes of the Na_V_. Accurate determination of the molecular determinants that confer the specificity of the μ-conotoxin PIIIA to different Na_V_ subtypes is essential for further engineering the specificity of the μ-conotoxins for therapeutic purposes.

### 1.1. Function of Na_V_

Voltage-gated sodium (Na_V_) channels play important roles in cell excitability and mediation of the ionic conductance through the cells [1,2,3]. Members of the Na_V_ channel family are classified as Na_V_1.1–1.9 according to the α subunit sequences [4]. Different Na_V_ subtypes usually distribute in different areas and have distinctive functions. Na_V_1.1, 1.2, 1.3 and 1.6 mainly exist in the central and peripheral nervous systems, while the Na_V_1.7, 1.8 and 1.9 are wildly distributed in peripheral sensory neurons [5,6]. The Na_V_1.4 is mainly existing in skeletal muscle [5,6], and Na_V_1.5 is essentially distributed in the cardiac muscle [5,6]. The Na_V_1.7, 1.8 and 1.9 are associated with neuropathic pain, and are considered as promising analgesic targets with a minimal side effect profile [5,6].

### 1.2. Structure of the Na_V_

**Figure 1:**
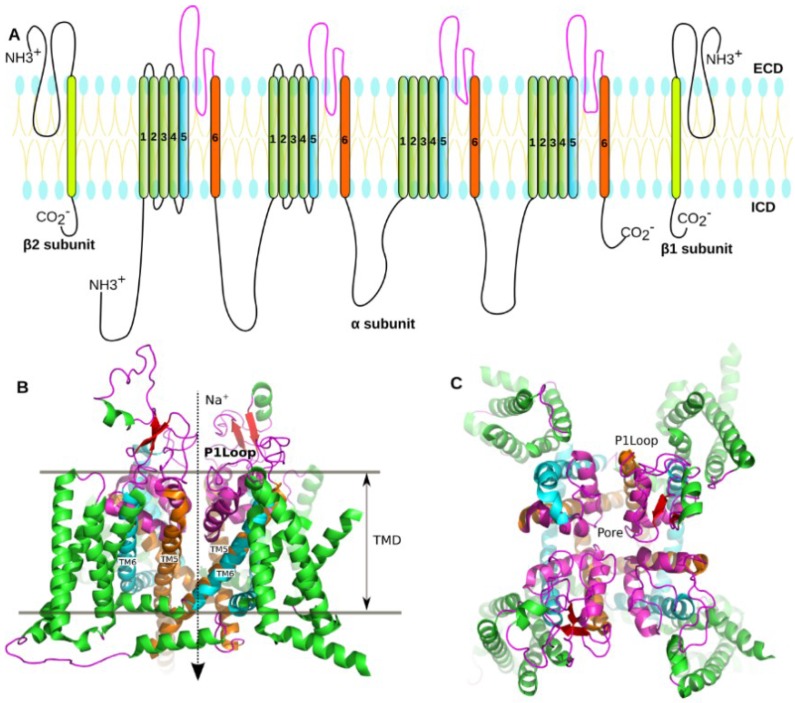
**Structure of the voltage-gated sodium channel.** (**A**) The topology of the eukaryotic Na_V_ channel. The voltage-sensing domains (VSDs) comprise four repeated segments (1–4) were colored in green. The segments 5 and 6, as well as the sequence between TM5 and TM6 (P1 loop) (Display in the **B**), that enclose to form the pore domain of the NaV were colored in cyan, orange, and magenta, respectively; (**B**) The cryo-EM structure of a eukaryotic Na_V_ channel (PDB code: 5X0M). The structure contains four voltage-sensing domains and one pore domain. TMD represents transmembrane domain, while TM5 and TM6 represent the segments 5 and 6 of transmembrane domain; (**C**) Crystal structure of the prokaryotic sodium channel from *Magnetococcus marinus* (NaVMs) (PDB code: 4OXS). The structure of the Na_V_Ms was colored according to the topology of the eukaryotic Na_V_ channel.

The eukaryotic Na_V_ channels comprise a pore-forming α subunit and one or two β subunits (Figure 1A). The α-subunit controls sodium ion selection, voltage sensing and inactivation, while the β subunit that includes an N-terminal extracellular immunoglobulin-like fold, a transmembrane segment, and intracellular fragments that help regulate channel dynamics, such as activation and inactivation of voltage dependence. The α subunit is a single chain with about 260 kDa in size, and it folds into four homologous repeats (domains I to IV), each containing six transmembrane segments, S1 to S6 (Figure 1). The central pore of Na_V_ is enclosed by S5 and S6 segments, and the selectivity filter (SF) of the pore is constituted of the intervening sequences of S5 and S6 (Figure 1B,C). The cryogenic electron microscopy (cryo-EM) structure of a putative Na_V_ channel from American cockroach (designated Na_V_PaS) at 3.8 angstrom resolution [7] (Figure 1B) as well as the cryo-electron microscopy (cryo-EM) structure of Ee Na_V_1.4 from the electric eel [8] give more detailed description of the structure composition of the Na_V_ channels.

### 1.3. Conotoxins

**Figure 2:**
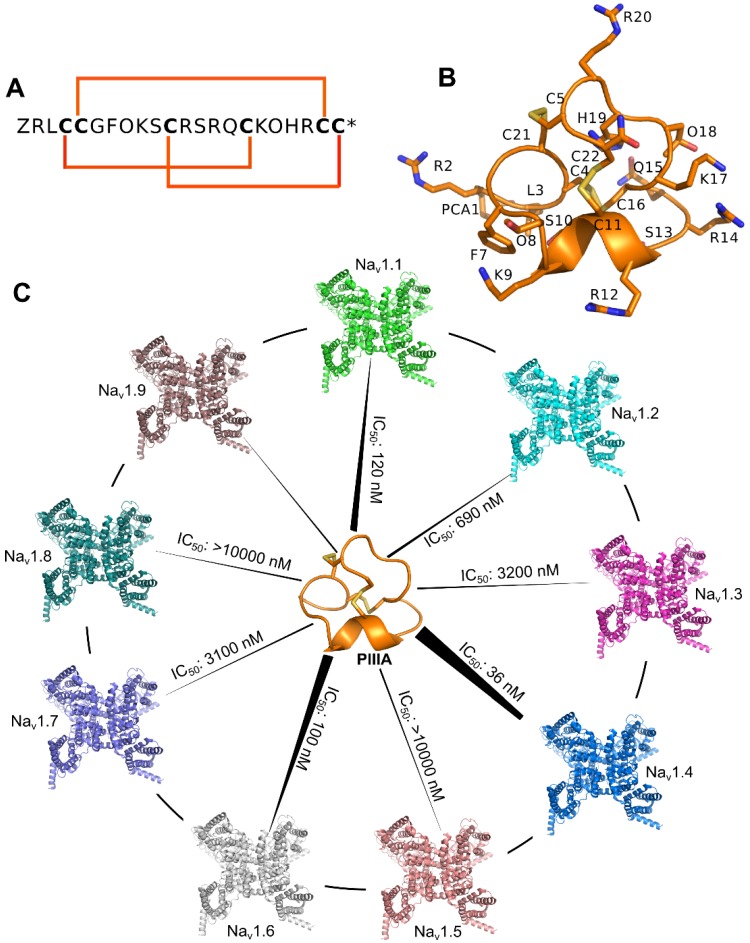
**Sequence, structure and function of μ-conotoxin PIIIA**. (**A**) Sequence of the PIIIA, the 6 bold characters “**C**” represent cysteine residues allowing the formation of three disulfide bonds; (**B**) Nuclear magnetic resonance (NMR) structure of the PIIIA (PDB Code: 1R9I); (**C**) Varied specificity of PIIIA to different subtypes of the Na_V_.

Conotoxins are short disulfide-rich peptide toxins comprising 12–30 amino acid residues and possess varied selectivity on ion channels [9]. μ-Conotoxins are selective and active blockers of the voltage-gated sodium channels. The μ-conotoxin PIIIA was isolated from *Conus purpurascens*, an Eastern Pacific fish hunting species of cone snails [10]. The PIIIA possesses varied potency towards TTX sensitive Na_V_ channels [11,12], and therefore the PIIIA serves as a valuable probe to distinguish Na_V_ channel subtypes when used in conjunction with other selective toxins [11,13] (Figure 2).

Without the high-resolution crystal structure of the eukaryotic voltage-gated sodium channel, crystal structures of the prokaryotic sodium channel Na_V_Ab have been widely used as templates to model the interactions between μ-conotoxins and Na_V_1.4 [14,15]. These models were used to predict the binding affinities of μ-conotoxins against Na_V_1.4, and explained their interaction mechanism [14,15]. However, the molecular determinants that confer the specificity of the μ-conotoxins to varied Na_V_ subtypes are still elusive, and no effective computational methodologies are available to predict the specificity of the μ-conotoxins.

In this study, the molecular determinants that confer the specificity of PIIIA to varied subtypes (hNa_V_1.1–1.9) of the Na_V_ were determined through extensive molecular dynamics simulations and binding energy calculations. And a computational method that could be used to predict the specificity of the μ-conotoxin PIIIA against different Na_V_ subtypes was proposed.

## 2. Methods

### 2.1. Flowchart for Specificity Prediction

Despite millions of years of divergent evolution between the Na_V_Ms and eukaryotic sodium channel, they both still possess similar pharmacological profiles to the channel blockers as shown by the functional electrophysiological studies [16]. Indeed, previous computational modeling studies suggested that crystal structure of NaChBac (Na(+)-selective channel of bacteria) could be used as template to model the pore domain of the human or rat Na_V_1.4 due to the relatively high sequence identity and structural similarity at the SF domain of the channel. In this study, the crystal structure of the Na_V_ Ms in open (PDB code 4CBC) was used as template to model the pore domain of the human Na_V_1.4. After building the homology model of Na_V_1.4, the NMR structure of the PIIIA (PDB code 1R9I) was docked into the extracellular pore domain of the Na_V_ 1.4 model [17]. Ten conformations of the PIIIA with minimum docking score were maintained and subjected to further molecular dynamics (MD) refinement and binding energy calculations using MMGB/SA method. The Molecular Mechanics/Poisson Boltzmann Surface Area (MM/PBSA) and Molecular Mechanics/Generalized Born Surface Area (MM/GBSA) approaches are more computationally efficient which estimate protein-protein binding free energies. Conformations of the PIIIA at Na_V_ 1.4 were ranked based on the binding energies calculated using MMGB/SA. And relatively higher energy conformations of the PIIIA at Na_V_1.4 were discarded, whereas the low energy conformations were maintained and further validated based on the known mutagenesis data [10,11]. Once the determined binding mode of PIIIA prokaryotic Na_V_1.4 was validated by the experimental data, it could be used as template to model interactions of PIIIA with other Na_V_ subtypes.

**Figure 3:**
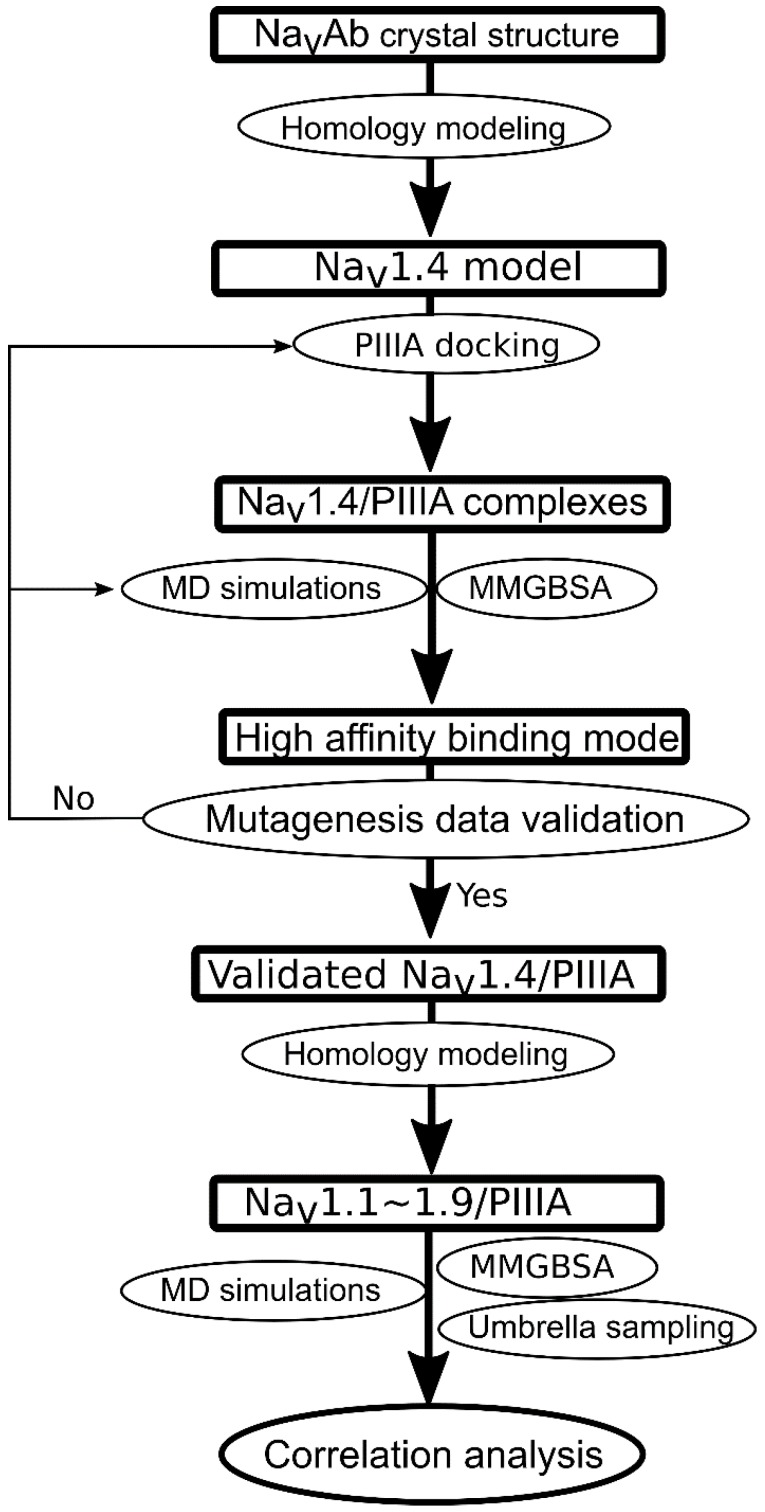
Flowchart for prediction of the specificity of the μ-conotoxin PIIIA to varied Na_V_ subtypes.

The binding modes of PIIIA against Na_V_1.x (x = 1, 2, 3, 5, 6, 7, 8, 9) were determined based on homology modeling using PIIIA/Na_V_1.4 as the template. The produced models were further refined using MD simulations. The binding energies of PIIIA against Na_V_1.x (x = 1, 2, 3, 5, 6, 7, 8, 9) were calculated using MMGB/SA method and umbrella sampling. The final step is the analysis of the correlation between the calculated and experimental derived binding affinities. The flowchart for PIIIA specificity prediction was summarized in Figure 3.

### 2.2. Homology Modeling of the Na_V_1.4

Models of the Na_V_1.4 were built using the crystal structure of prokaryotic Na_V_Ms channel (PDB code: 4P9O) as template in MODELLER (version 9v12) [18,19], as described previously [20]. The sequence of Na_V_1.4 was retrieved from the UniProt database [21], and was divided into four homologue sequence fragments based on the sequence of prokaryotic Na_V_Ms. The sequence alignment between the four homologue sequence fragments of Na_V_1.4 and sequence of the prokaryotic Na_V_Ms (Figure S1) was used to build 100 homology models of the Na_V_1.4, and the model with the lowest dope score [22] was selected for PIIIA docking.

### 2.3. PIIIA Docking

Docking the NMR structure of PIIIA (PDB Code: 1R9I) to Na_V_1.4 model was performed using AutoDock 4.2 [23]. Gasteiger charges were used, and nonpolar hydrogens of the macromolecule and ligand were merged. A grid box with dimensions of 60 Å × 60 Å × 60 Å and a grid spacing of 0.375 Å was set up and centered on the geometry center of the “P1 loop” of the four domains. Docking was performed using a Lamarckian genetic algorithm (LGA), with the receptor treated as rigid, the docking results were analyzed by AutoDock Tools. The produced conformations with top 10 docking score were selected for the MD refinement.

### 2.4. Restraint Molecular Dynamics Simulations

The selected 10 Na_V_1.4/PIIIA complexes were subjected to MD simulations for refinement of the PIIIA binding mode in AMBER 16 [24]. The MD simulations are performed using the ff14SB force field for the proteins and the peptide [25]. Parameters for the pyroglutamic acid (PCA) residue were prepared using the antechamber module in AMBER 16. Atom partial charges for PCA (pyroglutamic acid) were produced using the R.E.D Tools [26]. The PCA was incorporated into the PIIIA peptide as an unnatural amino acid. After building the whole system in LEaP that is a module of AMBER16, minimization was performed to remove the van der Waals clashes between PIIIA and Na_V_1.4. The 2000 steps of steepest descent minimization and 3000 conjugate gradient minimum were performed with the solute restrained using a harmonic force potential with a spring constant of 100 kcal/mol^−1^/Å^−2^. After the first round of minimization, the restraints were withdrawn and the whole system was minimized using the same parameters as above. MD simulations were carried out after minimization. First, the whole system was gradually heated from 50 K to 300 K at constant volume and temperature ensemble for 100 ps with the solute restrained with a harmonic force of 5 kcal/mol^−1^/Å^−2^. The simulations were then switched to constant pressure and temperature ensemble by maintaining the harmonic restraints in 100 ps. For the production run, the restraints on PIIIA as well as the P1 loop were withdrawn and 20 ns MD simulations were performed with the temperature and pressure maintained at 300 K and 1 bar, respectively. All bonds involving hydrogen atoms were constrained with the SHAKE algorithm with a time step of 2 fs [27]. The particle-mesh Ewald (PME) method was used to model long-range electrostatic interactions [28]. The following MD simulations on PIIIA/ Na_V_1.x (x = 1, 2, 3, 5, 6, 7, 8, 9) used the same parameters setting up as PIIIA/Na_V_1.4 system.

### 2.5. Molecular Dynamics Simulation of the Na_V_1.4 in Membrane

For evaluation of the membrane influences on the conformation of the Na_V_1.4 and the bound PIIIA, MD simulations were performed on Na_V_1.4 in *apo* and in complex with PIIIA. The Na_V_1.4 in *apo* or bound with PIIIA were inserted a bilayer containing a 2:2:1 mixture of POPC (1-palmitoyl-2-oleoyl-sn-glycero-3-phosphocholine):POPE (1-palmitoyl-2-oleoyl-sn-glycero-3-phosphoethanolamine):Cholesterol as suggested in previous study [29] with dimension of 80 × 80 × 80 Å^3^ and the system solvated with TIP3P [30] water molecules and Na^+^ and Cl^−^ ions such that the system was neutral with an overall concentration of 0.15 M in CHARMM-GUI that is a webserver for preparing MD simulation system (http://www.charmm-gui.org). The temperature of the system was gradually increased to 310 K and was equilibrated for 500 ps in NVT (constant-volume, constant-temperature) and NPT (constant-pressure, constant-temperature) ensembles, respectively, with the protein and lipids restrained with 10 kcal/mol/Å^2^ force. Langevin thermostat is used for the initial heating. For the second phase heating, an anisotropic Berendsen weak-coupling barostat is used to equilibrate the pressure in addition to the use of the Langevin thermostat to equilibrate the temperature. Then, the restraint of the membrane was withdrawn and the whole system was simulated for 20 ns in NPT for properly equilibrating the membrane system. And the restraint on the protein was gradually withdrawn in 10 steps in 5 ns MD simulations. Afterwards, 150 ns production run was performed on the Na_V_1.4 in *apo* and in complex with PIIIA, respectively. In production run, the temperature was controlled using the Langevin thermostat while pressure was controlled using the anisotropic Berendsen barostat. All simulations were performed using the Lipid14 force field [31] for the lipid, AMBER14SB force field for the protein [31]. The MD simulations used a time step of 2 fs and, all bonds involving hydrogen atoms were maintained to their standard length using the SHAKE algorithm [32]. Particle Mesh Ewald (PME) [33] was used with a cutoff of 10 Å for non-bonded atoms interactions and neighbor lists were updated every 10 steps.

### 2.6. Binding Energy Calculations Using MMGB/SA

Molecular mechanics generalized Born surface area (MMGB/SA) [34] were applied to calculate the binding affinities of PIIIA against the Na_V_ channels. The energies were averaged on the 50 frames extracted from the last 10 ns MD simulations. The parameters are as previously reported [20]. Briefly, the internal dielectric and external dielectric constants were set to 2.0 and 80.0, respectively. A probe radius of 1.4 Å, a grid spacing of 0.5 Å and ionic strength 0.1 mol/L were set up for the calculations.

### 2.7. Decomposition of the Binding Energies

The binding energies calculated using MMGB/SA were decomposed into energy contributions from each residue of the binding site using the MMPB/SA.py script in AMBER16. Parameters setting up was the same as above.

### 2.8. Binding Energies Calculations Using Umbrella Sampling

Umbrella sampling is one biased MD method that provides free energy along a reaction coordinate [35]. In umbrella sampling, a bias potential is applied to drive a system from one thermodynamic state to another along a reaction coordinate. Here, the distance between the center of mass of the PIIIA and the center of mass of the five symmetric CA atoms in the transmembrane domain were selected as the reaction coordinate, and along which a harmonic potential was added (Equation (1)).

(1) F(x) = k(x − x_0_)^2^

where the k is 100 kcal/mol/Å^2^ and x_0_ is the initial distance.

When the reaction coordinate (x − x_0_) reached 15 Å, the PIIIA was considered completely unbound from the channel. The reaction coordinate was divided into 30 windows, and in each window, 2 ns MD simulations were performed. The windows were then combined by the weighted histogram analysis method (WHAM) [36]. Conformations in the last 1.5 ns were used to derive the potential of mean force along the coordinate using WHAM. The binding/unbinding free energy of PIIIA was calculated using the PMF values at the end of unbinding.

## 3. Results and Discussion

### 3.1. Prediction of the Binding Mode of the PIIIA at Na_V_1.4

μ-Conotoxin PIIIA was positioned on the pore surface of the extracellular domain of Na_V_1.4 based on docking. During docking, the top 10 conformations were retained (Figure S2) for MD refinement and binding energy calculations. The PIIIA was positioned at the surface of the Na_V_1.4 with varied conformations (Figure S2A,B). The binding energies predicted using Autodock are considerably higher than the experimental values. The unfavorable binding energies given by the docking technique are originated from the poor accuracy when dealing with the flexibility of the protein and the peptide by AutoDock. During docking, only side chains of the PIIIA were considered as flexible, whereas side chains of the Na_V_1.4 were considered as rigid. As a consequence, the predicted binding affinities were underestimated using docking. Thus, it is prerequisite to refine the binding mode of PIIIA at Na_V_1.4 using MD, and re-rank the binding affinities of PIIIA at Na_V_1.4 using more rigorous energy estimation method, like MMGB/SA or MMPB/SA.

Here, the MMGB/SA method was used to calculate the binding affinities of the 10 conformations of PIIIA at Na_V_1.4 due to its slightly better performance than MMPB/SA method to rank the binding affinities of the α-conotoxin analogues in a previous study [20]. The ranking order for the 10 conformations significantly changed after re-ranking based on the MMGB/SA method (Table S1), and their backbone orientation at the binding site of Na_V_1.4 in some cases significantly varied (Figure S3). Conformation for the side chains of PIIIA varied significantly after MD simulations in most cases (not shown).

**Figure 4:**
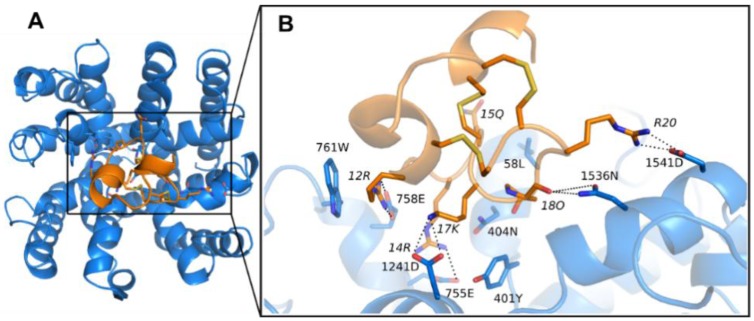
**The binding modes of μ-conotoxin PIIIA at Na_V_1.4.** The PIIIA and Na_V_1.4 were colored in orange and blue, respectively. The dashed lines show the hydrogen bonds or salt bridges. (**A**) The binding modes of Na_V_1.4/PIIIA; (**B**) The magnified show of the binding modes of Na_V_1.4/PIIIA in binding box.

Conformations of PIIIA with optimum binding energy were selected for accuracy validation by comparison with the mutagenesis data [37]. We found that the minimum binding energy conformation of PIIIA was the most consistent with the mutagenesis data, and its binding mode to Na_V_1.4 was shown in Figure 3. The PIIIA was positioned at the pore surface of the ECD with the axis of the helix between residue 6 and 15 tilted to the pore, resulting in the N-termini outward tilted from the binding site forming no direct interactions with residues of the binding site. The predicted binding mode of PIIIA is consistent with the previously determined binding mode of PIIIA at the Na_V_1.4 using restraint docking method [14]. The PIIIA is rich with positively charged residues, whereas the binding site of Na_V_1.4 possesses an abundance of negatively charged residues (Figure S4). Thus, the net charge of the PIIIA is overall complementary to the Na_V_1.4. Indeed, there are numerous salt-bridges formed at the interface between PIIIA and Na_V_ 1.4 (Figure 4). The R14 and K17 of PIIIA form salt-bridges with E755 and D1241, two of the filter residues at the pore domain, respectively. The R12 of PIIIA forms salt-bridges with E758 that is near the pore filter, while the R20 forms salt-bridges with D1541 at the periphery of the binding site. Besides, the hydroxyl group of O18 forms a pair of hydrogen bonds with N1536. Thus, our predicted binding mode of PIIIA against the Na_V_1.4 suggests that the salt-bridges as well as the hydrogen bonds are the dominant binding determinants for PIIIA binding to Na_V_1.4. Indeed, the previous mutagenesis data also supports that R14, R12, K17, and R20 contribute most to the binding affinity of PIIIA to Na_V_1.4 [37]. More detailed pairwise interactions between PIIIA and Na_V_1.4 were listed in Table 1.

### 3.2. Influences of the Membrane to the Conformation of the Na_V_1.4 in apo and Na_V_1.4 in Complex with PIIIA

For consideration of the influences of the membrane to the conformation of Na_V_1.4 and PIIIA/Na_V_1.4 complex model, nonrestraint molecular dynamic simulations were performed on the Na_V_1.4 model in *apo* or in complex with PIIIA embedded in the membrane. MD simulations results suggest that conformation of the Na_V_1.4 model in *apo* and in complex with PIIIA is stable in 100 ns MD. The RMSD (root-mean-square deviation) value of the Na_V_1.4 model in complex with PIIIA is lower than that in *apo* (Figure S5A). Obviously, binding PIIIA to Na_V_1.4 has a stabilization effect on this model. After 150 ns of non-restraint MD the binding mode of PIIIA at Na_V_1.4 is essentially the same to that determined using restraint MD without adding the membrane (Figure S5B). Thus, it is applicable to use more efficient restraint MD for the refinement of the binding mode of PIIIA at Na_V_1.4 without incorporation of the membrane.

### 3.3. Binding Modes of PIIIA at Other Subtypes of Na_V_

PIIIA possesses broad inhibitory spectra on Na_V_, from Na_V_1.1 to Na_V_1.8 [10,11,12,38,39,40]. Residues at the binding site of the α subunit of Na_V_ is highly conserved, and it might be applicable to homology model the binding modes of PIIIA to Na_V_1.x (x = 1, 2, 3, 5, 6, 7, 8, 9) by using PIIIA/Na_V_1.4 as the template. In this study, the PIIIA was directly modelled into the binding site of Na_V_1.x (x = 1, 2, 3, 5, 6, 7, 8, 9) using PIIIA/Na_V_1.4 as a template instead of using the docking method in considering of its incapacity for dealing with the flexibility of the protein and the peptide and its inaccuracy for prediction the binding affinity of the peptide ligands. Then the binding modes of PIIIA to Na_V_1.x (x = 1, 2, 3, 5, 6, 7, 8, 9) were refined using MD simulations.

**Figure 5:**
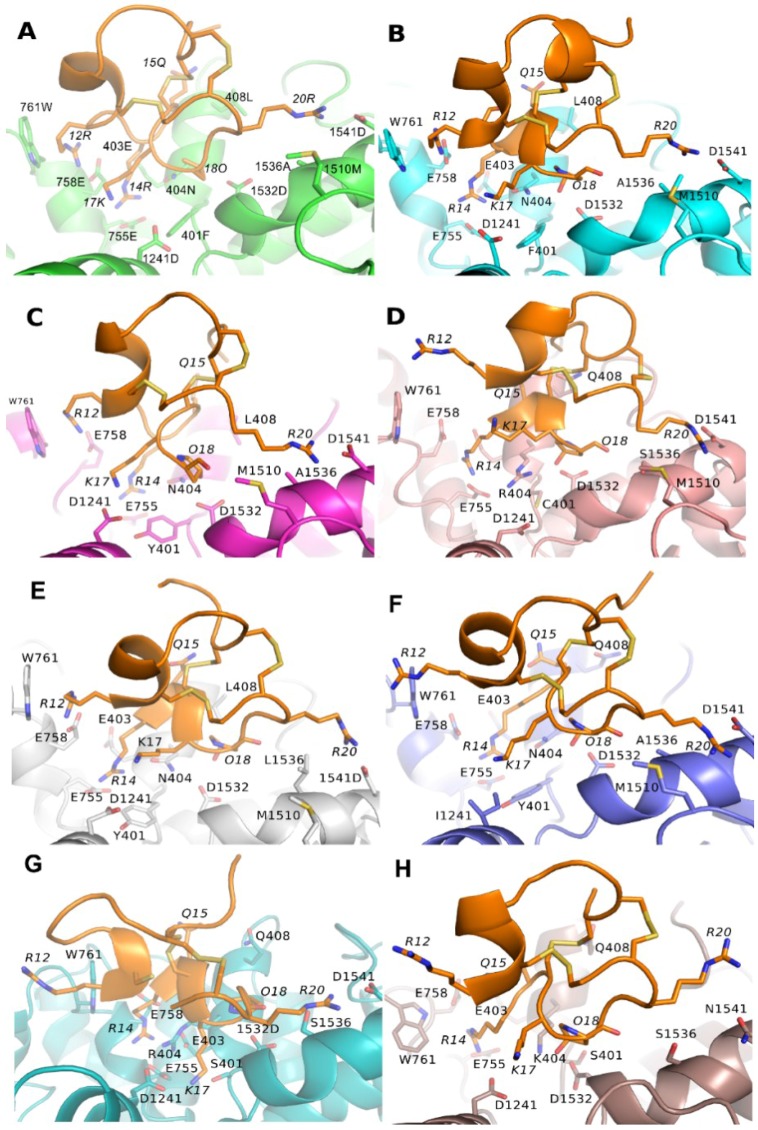
**Binding modes of PIIIA at different Na_V_ subtypes.** The PIIIA was shown in orange, and binding site of varied Na_V_ subtype from Na_V_1.1 to Na_V_1.9 was colored in green (Na_V_1.1), cyan (Na_V_1.2), magenta (Na_V_1.3), light pink (Na_V_1.5), gray (Na_V_1.6), blue (Na_V_1.7), teal (Na_V_1.8) and chocolate (Na_V_1.9). Residues at PIIIA were labeled in italics. For clarity, the hydrogen bonds were not shown. (**A**) Binding modes of Na_V_1.1/PIIIA; (**B**) Binding modes of Na_V_1.2/PIIIA; (**C**) Binding modes of Na_V_1.3/PIIIA ; (**D**) Binding modes of Na_V_1.5/PIIIA; (**E**) Binding modes of Na_V_1.6/PIIIA; (**F**) Binding modes of Na_V_1.7/PIIIA; (**G**) Binding modes of Na_V_1.8/PIIIA; (**H**) Binding modes of Na_V_1.9/PIIIA.

Binding modes of PIIIA to Na_V_1.x (x = 1, 2, 3, 5, 6, 7, 8, 9) are essentially the same to Na_V_1.4 (Figure 5). However, some of the key pairwise interactions at PIIIA/Na_V_1.4 are absent at PIIIA/Na_V_1.x (x = 2, 3, 5, 7, 8), whereas they are all conserved at PIIIA/Na_V_1.x (x = 1, 6). Because of presence of all these key pairwise interactions at PIIIA/Na_V_1.x (x = 1, 6), the PIIIA possesses comparable inhibitory activity between Na_V_1.x (x = 1, 6) and Na_V_1.4. By contrast, one to three of the key pairwise interactions were absent at PIIIA/Na_V_1.x (x = 2, 3, 5, 7, 8), which might explain the insensitivity of these Na_V_ subtypes to PIIIA. More detailed pairwise interactions between PIIIA and Na_V_1.x (x= 1, 2, 3, 5, 6, 7, 8, 9) are shown in Table 1.

### 3.4. Prediction of the Specificity of PIIIA to Varied NaV Subtypes

Prediction of the specificity of PIIIA to different Na_V_ subtypes presents a challenge, as it involves in differentiation of the binding affinity of PIIIA to several highly conserved Na_V_ subtypes. In this study, we used two computational methods, MMGB/SA and umbrella sampling to predict the binding affinities of PIIIA to Na_V_ subtypes from Na_V_1.1 to Na_V_1.9. 

**Figure 6:**
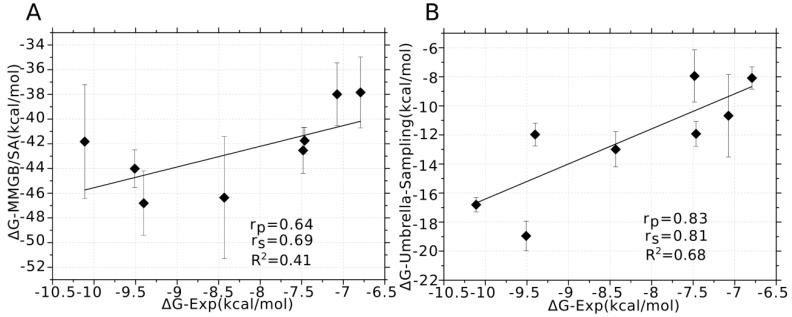
**Correlation between the binding energies derived from experiment and calculated using MM/GBSA and umbrella sampling.** (**A**) The binding affinity of PIIIA to varied Na_V_ subtypes was calculated using MMGB/SA method based on three MD trajectories of the top three models; (**B**) the binding affinity of PIIIA to varied Na_V_ subtypes was calculated using umbrella sampling on the top three models. The Pearson relation coefficient (r_p_) and Spearman relation coefficient (r_s_) were used to characterize the correlation between the calculated binding affinities and experimentally derived values.

The MMGB/SA was used to predict the binding affinities of PIIIA to varied Na_V_ subtypes due to its better performance than MMPB/SA at ranking the binding affinities of α-conotoxin analogues in our previous study [21]. For sufficiently sampling the conformation of PIIIA at varied Na_V_ subtypes, the top three DOPE score PIIIA/ Na_V_1.x (x = 1, 2, 3, 5, 6, 7, 8, 9) models were selected for MD simulations, and 20 ns MD simulations were performed on each of the PIIIA/Na_V_1.4 systems. Frames from the last 10 ns MD were used to calculate the binding energy using MMGB/SA method. The calculated binding affinities were averaged on the three MD trajectories for each of PIIIA/Na_V_1.x (x = 1, 2, 3, 4, 5, 6, 7, 8, 9) systems. In a previous study, we found that incorporation of the entropy component to the G-free energy gave slightly worse ranking of the binding affinity of the α-conotoxin analogues despite of the significantly increased consumption of the computational resources [21]. Thus, only the enthalpy rather than the entropy was calculated and used for ranking the binding affinity of PIIIA to varied Na_V_ subtypes. As shown by Table 2, the predicted binding energies without entropy using MMGB/SA are much lower than the experimental values, whereas they are modestly correlated with the experimental values giving a correlation coefficient of 0.41 (R^2^) (Figure 6). Thus, MD simulations coupling of MMGB/SA energy calculation could give modestly accurate predictions of the specificity of PIIIA to varied Na_V_ subtypes.

The umbrella sampling is more efficient than MD simulations for conformation sampling, and it has been frequently applied to predict the binding free energies of the conotoxins to their target proteins [41,42,43,44,45,46]. In this study, umbrella sampling was used to predict the binding affinities of PIIIA against varied Na_V_ subtypes. The above MD refined the top three models of the PIIIA/Na_V_ systems, and were used as the initial for umbrella sampling simulations. The predicted binding free energies of PIIIA against varied Na_V_ subtypes were derived from the calculation of potential of mean force (PMF) and were shown in Table 2. The absolute values of the predicted binding free energies using umbrella sampling are quite similar to the experimental values, and are high correlated to the experimental values with a correlation efficient of 0.68 (R^2^). Thus, umbrella sampling could give a highly accurate ranking of the inhibition activity of PIIIA to different Na_V_ subtypes. Umbrella sampling gave a better prediction of the specificity of PIIIA to varied Na_V_ subtypes due to its more efficient conformation sampling. 

There is no binding or IC_50_ data for PIIIA to Na_V_1.9. Binding energies calculation from both MMGB/SA and umbrella sampling together predict that PIIIA possesses poor binding affinity to the Na_V_1.9.

### 3.5. Binding Energy Decomposition Using MMGB/SA

**Figure 7:**
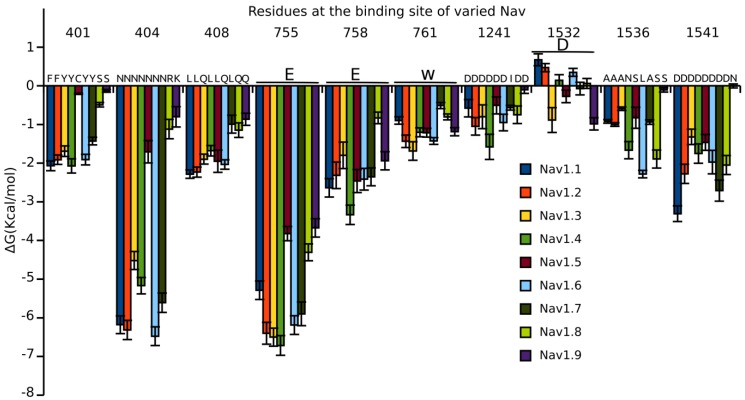
**Energetic contribution of the residues at the binding site of varied Na_V_ to the binding affinity of PIIIA**. Residues at position 755, 758, 761, 1532 are conserved and are shown in bold. Residue side chain energetic contribution to the binding affinity of PIIIA to varied Na_V_ subtypes from Na_V_1.1 to Na_V_1.9 are shown in different colors.

The binding energy calculated using MMGB/SA was decomposed into energetic contributions from each residue of the binding site (Figure 7 and Table S2). Binding energy decomposition suggests that all residues of the binding site except for D1532 significantly contributes to the binding affinity of PIIIA to Na_V_. Binding mode analysis suggests that the D1532 locates in the binding site forming van der Waals contacts with the backbone of the PIIIA (Figure 4). The high desolvation energy of D1532 might result in its unfavorable binding to most of the Na_V_ subtypes, except for Na_V_1.3 in which the D1532 forms an unstable hydrogen bond with PIIIA O18. An aromatic residue such as Y or F at 401 significantly contributes to the binding affinity of PIIIA to Na_V_1.1, Na_V_1.2, Na_V_1.3, Na_V_1.4, Na_V_1.6 and Na_V_1.7. In our model, the side chain of the aromatic residue at 401 stacks with the side chain of K17, forming favorable van der Waals contacts (Figure 4). By contrast, the C and S merely form few contacts with the side chain of K17, explaining their little energetic contribution to the binding affinity of PIIIA to Na_V_1.5, Na_V_1.8 and Na_V_1.9. A neutral N at 404 is more energetically favorable for the binding of PIIIA to Na_V_1.x (x = 1, 2, 3, 4, 6, 7) than the positively charged R or K of Na_V_1.x (x = 5, 8, 9). Binding mode analysis indicates that side chain of N can form a hydrogen bond with R14, whereas this hydrogen bond is absent when a positively charged residue is present at 404 (Figure 4). The L and Q at 408 showed only a little difference to the binding affinity of PIIIA to varied subtypes of Na_V_, despite of their side chain hydrophobicity difference. The L and Q at 408 are at the periphery of the binding site where they both form few van der Waals contacts with the PIIIA explaining their little difference to the binding affinity of PIIIA. The conserved E755 is located at the center of the binding site, substantially contributing to the binding affinity of PIIIA by forming salt bridges with the side chain of R14 with energetic contribution varied from −4 kcal/mol to −7 kcal/mol (Figure 5). By contrast, the conserved E758 contributes less than E755 to the binding affinity of PIIIA to subtypes of Na_V_ by forming an electrostatic interaction with R12 of PIIIA. A conserved aromatic residue, W761, contributes −2~−1 kcal/mol binding energy to PIIIA by forming van der Waal contacts with side chain of R12. A D at 1241 is conserved among varied subtypes of Na_V_1 except for Na_V_1.7, on which an I is present this position. The D1241 locates at the periphery of the binding site where it only contributes −2~0 kcal/mol binding energy to the total binding affinity of PIIIA. The side chain of K17 on PIIIA tilted to the D1241 and could form favorable electrostatic interaction in MD. The D at 1532 is conserved across all Na_V_ subtypes. The D1532 contributes little or even unfavorable binding energy to the binding affinity of PIIIA to different subtypes of Na_V_1 except for to the Na_V_1.3 in which D1532 can form a hydrogen bond with the hydroxyl group of O18. Residues at 1536 are not conserved across different subtypes of Na_V_. An N at 1536 of Na_V_1.4 can form a hydrogen bond with the hydroxyl group of O18, whereas this hydrogen bond is absent when A, S or L are present at the same position. The side chain of L1536 at Na_V_1.6 stacks with the side chain of R20 as well as the backbone of PIIIA, and significantly contributes to its binding affinity. A S at 1536 can form hydrogen bond with R20 and significantly contributes to its binding to Na_V_1.8, whereas such pairwise interaction is absent at the Na_V_1.9. Thus, the same residue at the same positions of different subtypes of Na_V_ might play different roles to the binding affinity of PIIIA. The D at 1541 is conserved across all Na_V_ subtypes from Na_V_1.1 to Na_V_1.8, whereas an N is at the same position of Na_V_1.9. Either an D or an N at 1541 of Na_V_ subtypes contributes to about −2 kcal/mol energy to the binding affinity of PIIIA by forming salt bridges or hydrogen bonds with R20 of PIIIA.

Overall, the binding site of Na_V_ is rich of negatively charged residues such as D and E, and thus the binding site shows an overall negative electrostatic potential (Figure S4). By contrast, the PIIIA contains six positively charged residues and shows the positive electrostatic potential that is complementary to that of the Na_V_ (Figure S4). Thus, the long range electrostatic interaction can be the driving force for the binding of PIIIA to the Na_V_ at long range distance. At short range distance, the pairwise electrostatic interactions and hydrogen bond interactions contribute most to the binding affinity of PIIIA to Na_V_ subtypes. Binding energy decomposition clearly shows that the conserved residues and the non-conserved residues have synergistic effects to affect the binding affinity of PIIIA to Na_V_ subtypes.

## 4. Conclusions

A computational method combining homology modeling, docking, molecular dynamics simulations and binding energy calculations was established and successfully applied to determine the specificity of PIIIA to varied Na_V_ subtypes from Na_V_1.1 to Na_V_1.9. The predicted binding free energies using umbrella sampling and MMGB/SA were correlated with experimental values giving a correlation coefficient (R^2^) of 0.68 and 0.41, respectively. The performance of umbrella sampling is superior to MMGB/SA due to the more powerful conformation sampling of the former. Interestingly, the predicted binding affinities given by umbrella sampling is very similar to the experimental values.

Binding modes analysis identified nonconserved residues existing at the interface between PIIIA and Na_V_1.x (x = 1 to 9). These nonconserved residues formed nonconserved interactions at the interface between PIIIA and Na_V_ subtypes, which might confer the specificity of PIIIA. Indeed, results from binding energy decomposition showed that these nonconserved residues contributed varied binding energies to the binding affinity of PIIIA to varied Na_V_ subtypes. Intriguingly, we also found that even the conserved residues could contribute varied binding energies to the binding of PIIIA to varied Na_V_ subtypes. Therefore, the conserved and nonconserved residues might play synergistic effects together to influence the specificity of PIIIA to varied Na_V_ subtypes. Overall, results from this study are valuable for our understanding the molecular determinants that confer the specificity of PIIIA to varied Na_V_ subtypes and for further engineering the specificity of the μ-conotoxins to the interested Na_V_ subtypes.

## Figures and Tables

**Table 1:** Key pairwise interacting residues from PIIIA and binding site of the Na_V_1.1 to Na_V_1.9.

Na_V_	R12 ^a^	R14 ^a^	Q15 ^a^	K17 ^a^	O18 ^a^	R20 ^a^
**Na_V_1.1**	E758, W761	F401, N404, E755	L408	-	N404, D1533	A1536, D1541
**Na_V_1.2**	E758, W761	F401, N404, E758	L408	D1241	N404, D1533	A1536, D1541
**Na_V_1.3**	E758, W761	E755	L408	D1241	N404, D1533	A1536, D1541
**Na_V_1.4**	E758, W761	F401, N404, E755, E758	L408	D1241	N404, D1533, N1536	D1541
**Na_V_1.5**	W761	E755, E758	Q408	-	R404	D1541
**Na_V_1.6**	E758, W761	Y401, N404, E755	L408	-	R1533	L1536, D1541
**Na_V_1.7**	W761	F401, N404, E755, E758	Q408	-	N404, D1533	A1536, D1541
**Na_V_1.8**	W761	N404, E755	Q408	D1241, G1536	D1532, D1536	S1541
**Na_V_1.9**	W761	E755, E758	Q408	-	D1532, S1536	N1541

^a^ Residues of the Na_V_ within 4 Å from the key residues of PIIIA were listed in the table.

**Table 2:** Calculated binding affinities of PIIIA to varied Na_V_ subtypes from Na_V_1.1 to Na_V_1.9.

Na_V_	IC_50_	MMGB/SA ^a^	Umbrella Sampling ^b^	Experimental (kcal/mol) ^c^
Na_V_1.1	120 nm	−46.81 ± 2.61	−11.97 ± 0.78	−9.40
Na_V_1.2	620 nm	−46.36 ± 4.94	−12.99 ± 1.21	−8.43
Na_V_1.3	3.2 μm	−41.74 ± 1.08	−11.93 ± 0.87	−7.46
Na_V_1.4	36 nm	−41.82 ± 4.61	−16.81 ± 0.49	−10.11
Na_V_1.5	10 μm	−37.83 ± 2.88	−8.09 ± 1.44	−6.79
Na_V_1.6	100 nm	−44.01 ± 1.51	−18.95 ± 1.02	−9.51
Na_V_1.7	>10 μm	−42.53 ± 1.86	−7.94 ± 1.80	−7.48
Na_V_1.8	>10 μm	−38.00 ± 2.55	−10.68 ± 2.84	−7.07
Na_V_1.9	-	−29.24 ± 5.03	−9.41 ± 2.01	-

^a^ The binding affinities of PIIIA to varied Na_V_ subtypes were calculated using MMGB/SA method. Twenty MD simulations were performed on the top 3 PIIIA/Na_V_ models from homology modeling. The MMGB/SA calculation was performed on the last 10 ns of the five MD trajectories; ^b^ The binding affinities of PIIIA were calculated using umbrella sampling (US) on the top 3 PIIIA/Na_V_ models; ^c^ Binding affinities of PIIIA were derived from the IC_50_ of PIIIA to varied Na_V_ subtypes from Na_V_1.1 to Na_V_1.9 using ∆G = −RTln(IC_50_).

## Supplementary Materials

The following are available online at http://www.mdpi.com/1660-3397/16/5/153/s1.
