# Supplementary material for: Determination of the μ-Conotoxin PIIIA Specificity Against Voltage-Gated Sodium Channels from Binding Energy Calculations
Source: Mar Drugs. 2018 May 7;16(5):153. doi: 10.3390/md16050153 (PMC5983284; doi:10.3390/md16050153)
Supplement: Supplementary file 1 [file marinedrugs-16-00153-s001.pdf]

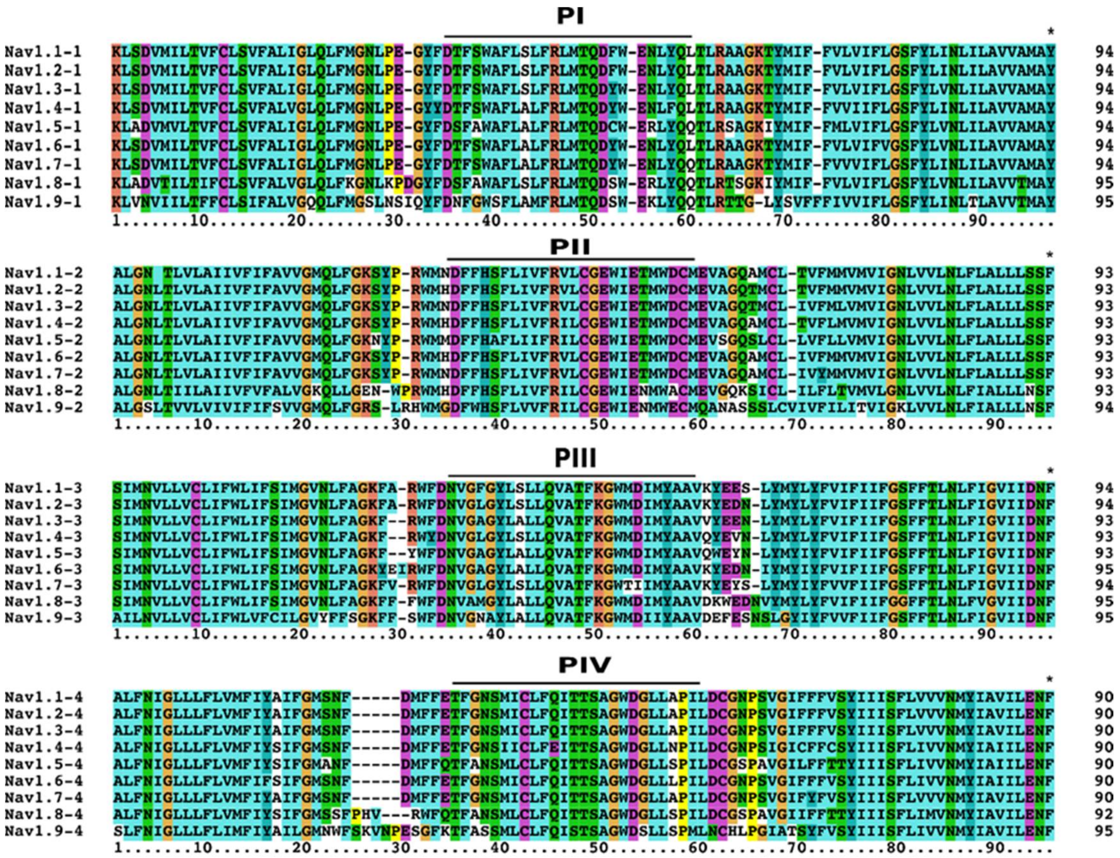

Figure S1. Sequence alignment between the four PIIIA binding domains of the varied Na_V subtypes. Loops with low sequence identify as well as the voltage sensing domain (VSD) were truncated from the sequence alignment. The P loop from the four domains were labelled as PI to PIV.

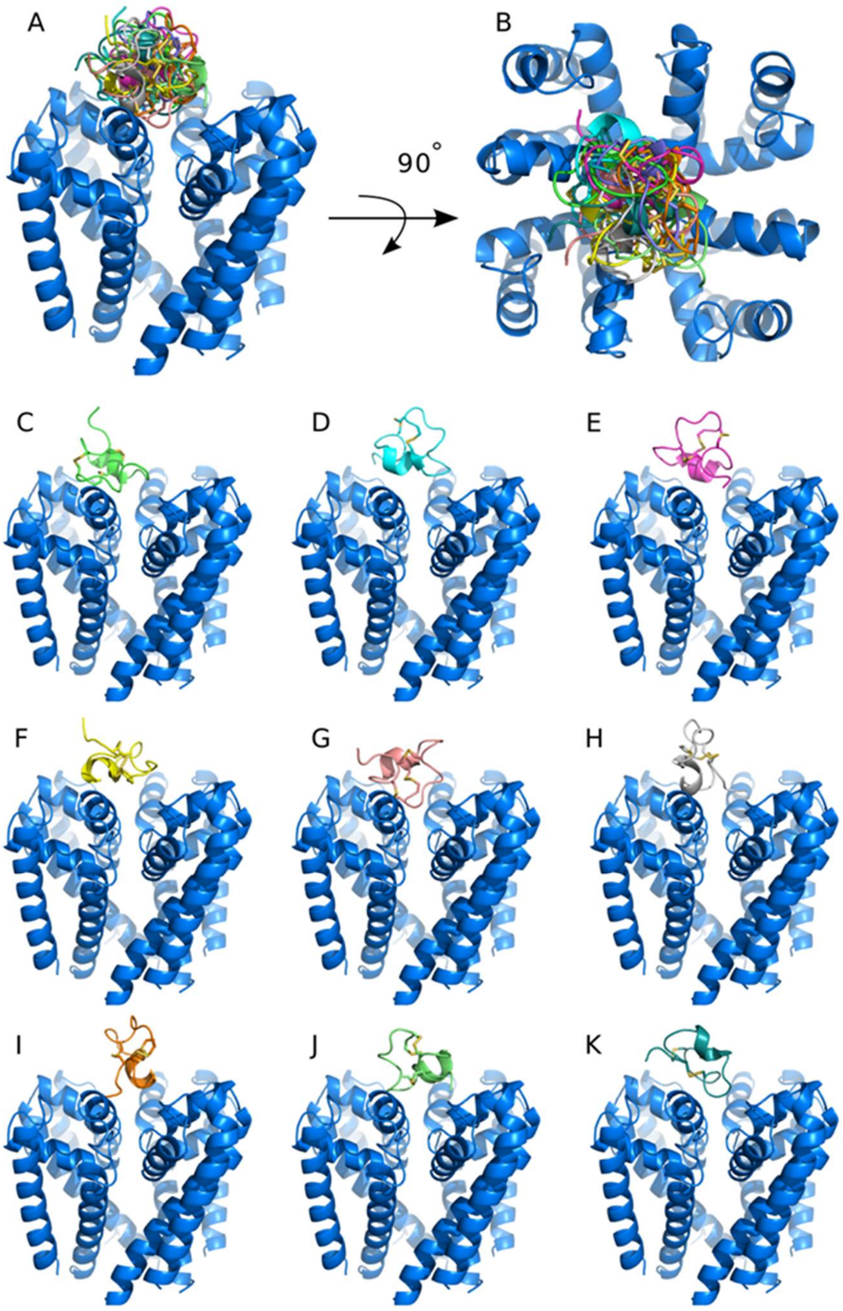

Figure S2. The top 10 conformations of PIIIA at the binding site of $Na_V1.4$ from docking in AutoDock. A) and B) show the top 10 conformations of PIIIA together at the binding site of $Na_V1.4$. Obviously, the top 10 conformations of PIIIA at the binding site are in varied orientations. C) – K) show the orientation of the top 10 conformations of PIIIA at the binding site of $Na_V1.4$, respectively.

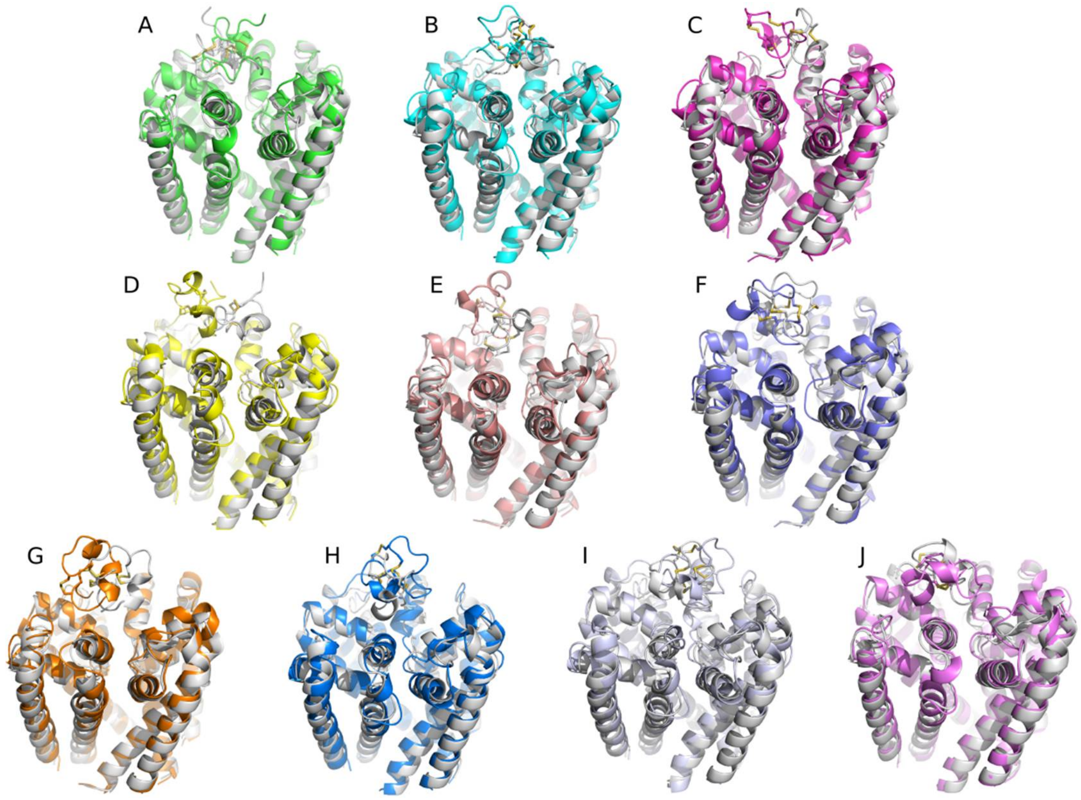

Figure S3. Binding modes of the top 10 conformations refined using MD. A) – J), Comparison of the MD refined 10 conformations of PIIIA bound with $Na_V1.4$ (in varied colors) with conformations prior to MD (in gray). MD refinement could result in significant conformation deviation from that determined using docking.

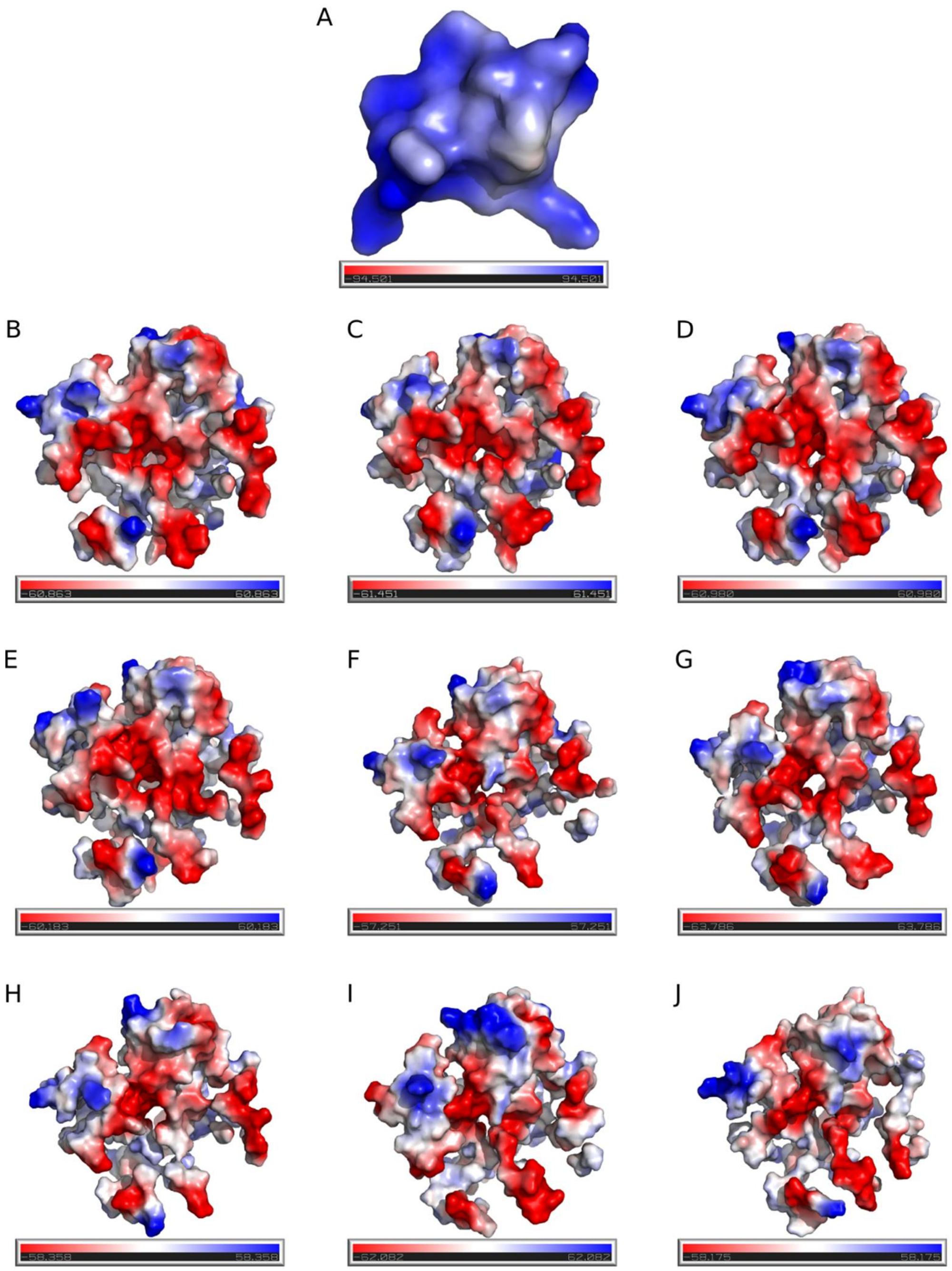

Figure S4. Surface electrostatic potential of PIIIA and Na$_V$ channels. A) shows the structure of PIIIA and five key residues on its surface; B) shows the electrostatic potential of PIIIA；C)−K), show the surface electrostatic potential of the pore domain/ or PIIIA binding site of Na$_V$ subtypes.

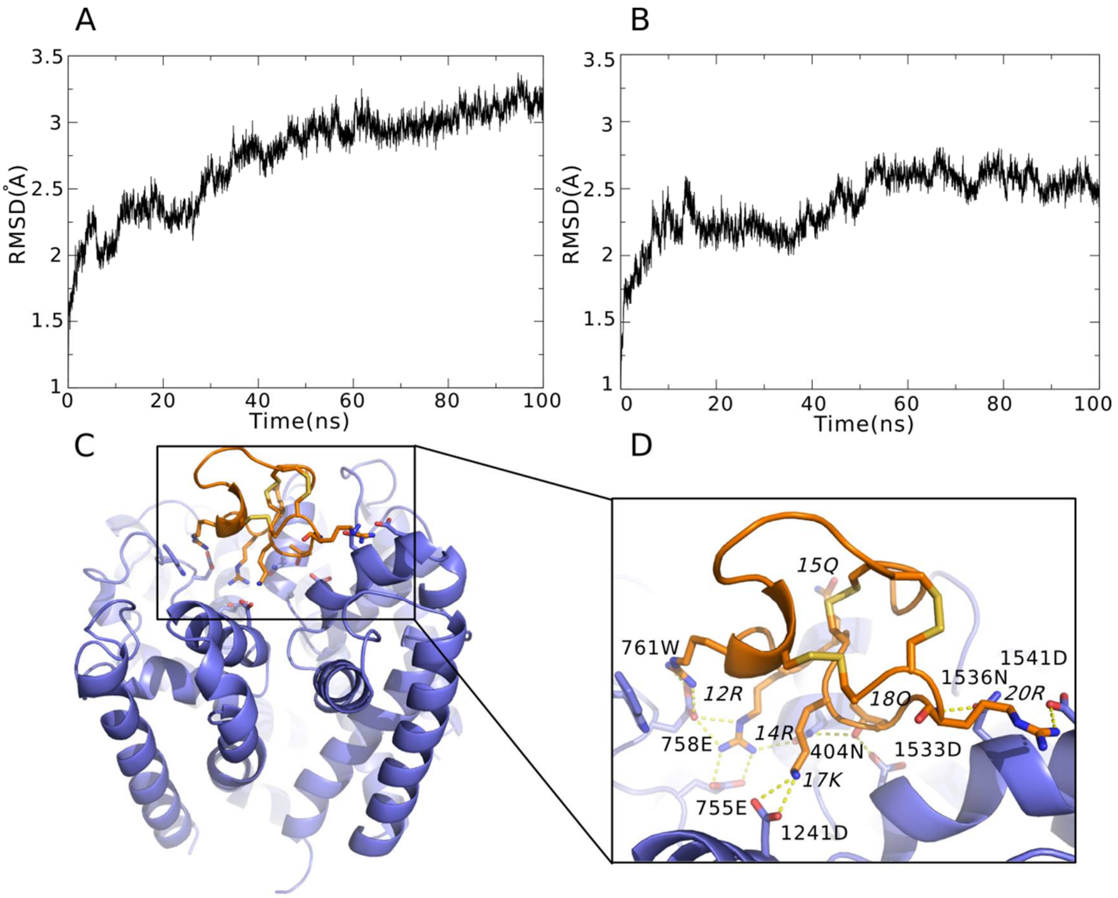

Figure S5. Conformation of the Na$_V$1.4 embedded in membrane in MD simulations. Root mean square deviation (RMSD) for the backbone of Na$_V$1.4 embedded in membrane in 100 ns non-restraint MD simulations. A),B) show the backbone RMSD of Na$_V$1.4 in *apo* and bound with PIIIA, respectively. C,D) show the binding mode of PIIIA to Na$_V$1.4. Residues at the PIIIA were labelled in italic.

Table S1. Energetic contribution for the side chain of the key residues at the binding site of Na_V subtypes.

Na_V	401[a]	404[a]	408[a]	755[a]	758[a]	761[a]	1241[a]	1532[a]	1536[a]	1541[a]
Na_V1.1	-2.07	-6.18	-2.29	-5.29	-2.64	-0.90	-0.58	0.67	-0.92	-3.31
Na_V1.2	-1.91	-6.32	-2.23	-6.40	-2.32	-1.44	-1.05	0.47	-1.00	-2.28
Na_V1.3	-1.69	-4.52	-1.90	-6.50	-1.80	-1.69	-0.80	-0.89	-0.59	-1.32
Na_V1.4	-2.07	-5.17	-1.68	-6.72	-3.34	-1.20	-1.58	0.14	-1.67	-1.75
Na_V1.5	-0.21	-1.71	-1.95	-3.82	-2.46	-1.21	-0.51	-0.27	-0.83	-1.46
Na_V1.6	-1.91	-6.48	-2.03	-6.19	-2.42	-1.43	-0.95	0.35	-2.28	-1.97
Na_V1.7	-1.43	-5.61	-0.99	-5.90	-2.35	-0.51	-0.57	-0.07	-0.94	-2.71
Na_V1.8	-0.50	-1.12	-1.15	-4.31	-0.83	-0.80	-0.77	0.06	-1.90	-2.05
Na_V1.9	-0.13	-0.80	-0.87	-3.67	-1.94	-1.19	-0.10	-0.98	-0.11	-0.01

[a] Side chain energetic contribution was calculated based on binding energy decomposition using MMGB/SA method.
